# Pre-diagnostic concordance with the WCRF/AICR guidelines and survival in European colorectal cancer patients: a cohort study

**DOI:** 10.1186/s12916-015-0332-5

**Published:** 2015-05-07

**Authors:** Dora Romaguera, Heather Ward, Petra A Wark, Anne-Claire Vergnaud, Petra H Peeters, Carla H van Gils, Pietro Ferrari, Veronika Fedirko, Mazda Jenab, Marie-Christine Boutron-Ruault, Laure Dossus, Laureen Dartois, Camilla Plambeck Hansen, Christina Catherine Dahm, Genevieve Buckland, María José Sánchez, Miren Dorronsoro, Carmen Navarro, Aurelio Barricarte, Timothy J Key, Antonia Trichopoulou, Christos Tsironis, Pagona Lagiou, Giovanna Masala, Valeria Pala, Rosario Tumino, Paolo Vineis, Salvatore Panico, H Bas Bueno-de-Mesquita, Peter D Siersema, Bodil Ohlsson, Karin Jirström, Maria Wennberg, Lena M Nilsson, Elisabete Weiderpass, Tilman Kühn, Verena Katzke, Kay-Tee Khaw, Nick J Wareham, Anne Tjønneland, Heiner Boeing, José R Quirós, Marc J Gunter, Elio Riboli, Teresa Norat

**Affiliations:** Department of Epidemiology and Biostatistics, School of Public Health, Imperial College London, St. Mary’s Campus, Norfolk Place, W2 1PG London, UK; Instituto de Investigación Sanitaria de Palma (IdISPa), Hospital Universitario Son Espases, Research Unit I-1, Ctra. de Valldemossa 79, 07010 Palma de Mallorca, Spain; CIBER Fisiopatología de la Obesidad y Nutrición (CIBEROBN), Instituto de Salud Carlos III, C/ Monforte de Lemos 3-5. Pabellón 11. Planta 0. 28029, Madrid, Spain; Global eHealth Unit, Department of Primary Care and Public Health, School of Public Health, Imperial College London, The Reynolds Building, St Dunstan’s Road, London, W6 8RP UK; Department of Epidemiology, Julius Center for Health Sciences and Primary Care, University Medical Center Utrecht, P.O. Box 85500 3508 GA, Utrecht, The Netherlands; International Agency for Cancer Research (IARC), 150 Cours Albert Thomas, 69372, Lyon, CEDEX 08 France; Department of Epidemiology, Rollins School of Public Health, Emory University, 1518 Clifton Rd, Atlanta, GA USA; Winship Cancer Institute, Emory University, 1518 Clifton Rd, Atlanta, GA 30322 USA; Inserm (Institut National de la Santé et de la Recherche Médicale), Centre for Research in Epidemiology and Population Health (CESP), U1018, Team 9, 114 rue Édouard Vaillant, 94805 Villejuif, Cedex France; Univ Paris Sud, UMRS 1018, 114 rue Édouard Vaillant 94805, Villejuif, France; Gustave Roussy, 114 rue Édouard Vaillant 94805, Villejuif, France; Section for Epidemiology, Department of Public Health, Aarhus University, Bartholins Allé 2, DK-8000 Aarhus C, Denmark; Unit of Nutrition, Environment and Cancer, Cancer Epidemiology Research Programme, Catalan Institute of Oncology (ICO-IDIBELL), Avda. de la Granvia de l’Hospitalet, 199-203, 08908 Barcelona, Spain; Escuela Andaluza de Salud Pública, Instituto de Investigación Biosanitaria ibs. GRANADA, Hospitales Universitarios de Granada/Universidad de Granada, Campus Universitario de Cartuja, Cuesta del Observatorio, 4, 18011 Granada, Spain; CIBER de Epidemiología y Salud Pública (CIBERESP), Instituto de Salud Carlos III, C/ Monforte de Lemos 3-5. Pabellón 11. Planta 0. 28029, Madrid, Spain; Public Health Direction and Biodonostia Basque Regional Health Department, Avenida Navarra 4, 20013 San Sebastian, Spain; Department of Epidemiology, Murcia Regional Health Council, Ronda de Levante, 11, 30008 Murcia, Spain; Department of Health and Social Sciences, Universidad de Murcia, Campus Universitario de Espinardo, s/n, 30100 Espinardo, Murcia, Spain; Navarre Public Health Institute, C/ Leyre, 15 31003 Pamplona, Spain; Cancer Epidemiology Unit, Nuffield Department of Population Health, University of Oxford, Richard Doll Building, Roosevelt Drive, Oxford, OX3 7LF UK; Hellenic Health Foundation, 13 Kaisareias Street, Athens, GR-115 27 Greece; Bureau of Epidemiologic Research, Academy of Athens, 23 Alexandroupoleos Street, Athens, GR-115 27 Greece; Department of Hygiene, Epidemiology and Medical Statistics, University of Athens Medical School, 75 M. Asias Street, Goudi, GR-115 27, Athens, Greece; Department of Epidemiology, Harvard School of Public Health, 677 Huntington Avenue, Boston, MA 02115 USA; Molecular and Nutritional Epidemiology Unit, Cancer Research and Prevention Institute – ISPO, Via delle Oblate 4, 50141 Florence, Italy; Epidemiology and Prevention Unit, Fondazione IRCCS, Istituto Nazionale dei Tumori, Via Giacomo Venezian, 1, Milan, Italy; Cancer Registry and Histopathology Unit, “Civic – M.P. Arezzo” Hospital, Via Dante Alighieri 109, I-97100, ASP, Ragusa, Italy; HuGeF Foundation, via Nizza 52, Turin, Italy; Dipartimento di Medicina Clinica e Chirurgia, Federico II University, VIA PANSINI 5, 80131 Naples, Italy; Department for Determinants of Chronic Diseases (DCD), National Institute for Public Health and the Environment (RIVM), Antonie van Leeuwenhoeklaan 9, 3721 MA, Bilthoven / PO Box 1, 3720 BA Bilthoven, The Netherlands; Department of Gastroenterology and Hepatology, University Medical Centre, P.O. Box 85500, 3508 GA Utrecht, The Netherlands; Department of Social and Preventive Medicine, Faculty of Medicine, University of Malaya, Lembah Pantai, 50603 Kuala Lumpur, Malaysia; Division of Internal Medicine, Department of Clinical Sciences, Skane University Hospital, Malmo, Lund University, Inga-Maria Nilssons gata 32, 205 02 Lund, Sweden; Department of Clinical Sciences, Division of Oncology and Pathology, Lund University, Barngatan 2B, SE- 221 85 Lund, Sweden; Public Health and Clinical Medicine, Nutritional Research, Umeå University, S-901 87 Umeå, Sweden; Arctic Research Centre, Umeå University, S-901 87 Umeå, Sweden; Department of Community Medicine, Faculty of Health Sciences, University of Tromsø, The Arctic University of Norway, ISM - Universitetet i Tromsø, 9037 Tromsø, Norway; Department of Research, Cancer Registry of Norway, P.B. 5313, Majorstuen, 0304 Oslo Norway; Department of Medical Epidemiology and Biostatistics, Karolinska Institutet, PO Box 281, 171 77 Stockholm, Sweden; Department of Genetic Epidemiology, Folkhälsan Research Center, Folkhälsan Research Center, Biomedicum 1, Haartmansgatan 8, PB 63 (B336b), FI-00014 University of Helsinki, Helsinki, Finland; German Cancer Research Center (DKFZ), Division of Cancer Epidemiology Im Neuenheimer Feld 581, D-69120 Heidelberg, Germany; University of Cambridge School of Clinical Medicine, Clinical Gerontology Unit Box 251, Addenbrooke’s Hospital, Cambridge, CB2 2QQ UK; MRC Epidemiology Unit, University of Cambridge, Addenbrooke’s Hospital, Cambridge, CB2 2QQ UK; Danish Cancer Society Research Center, Strandboulevarden 49, 2100 Copenhagen, Denmark; Department of Epidemiology, German Institute of Human Nutrition Potsdam-Rehbruecke, Nuthetal, 69120 Germany; Public Health Directorate, Ciriaco Miguel Vigil 9, Oviedo, 33417 Asturias Spain

**Keywords:** Colorectal cancer, Diet, Healthy lifestyle, Physical activity, Survival, Weight

## Abstract

**Background:**

Cancer survivors are advised to follow lifestyle recommendations on diet, physical activity, and body fatness proposed by the World Cancer Research Fund/American Institute of Cancer Research (WCRF/AICR) for cancer prevention. Previous studies have demonstrated that higher concordance with these recommendations measured using an index score (the WCRF/AICR score) was associated with lower cancer incidence and mortality. The aim of this study was to evaluate the association between pre-diagnostic concordance with WCRF/AICR recommendations and mortality in colorectal cancer (CRC) patients.

**Methods:**

The association between the WCRF/AICR score (score range 0–6 in men and 0–7 in women; higher scores indicate greater concordance) assessed on average 6.4 years before diagnosis and CRC-specific (n = 872) and overall mortality (n = 1,113) was prospectively examined among 3,292 participants diagnosed with CRC in the European Prospective Investigation into Cancer and Nutrition (EPIC) cohort (mean follow-up time after diagnosis 4.2 years). Multivariable Cox proportional hazard models were used to estimate hazard ratios (HRs) and 95% confidence intervals (CIs) for mortality.

**Results:**

The HRs (95% CIs) for CRC-specific mortality among participants in the second (score range in men/women: 2.25–2.75/3.25–3.75), third (3–3.75/4–4.75), and fourth (4–6/5–7) categories of the score were 0.87 (0.72–1.06), 0.74 (0.61–0.90), and 0.70 (0.56–0.89), respectively (*P* for trend <0.0001), compared to participants with the lowest concordance with the recommendations (category 1 of the score: 0–2/0–3). Similar HRs for overall mortality were observed (*P* for trend 0.004). Meeting the recommendations on body fatness and plant food consumption were associated with improved survival among CRC cases in mutually adjusted models.

**Conclusions:**

Greater concordance with the WCRF/AICR recommendations on diet, physical activity, and body fatness prior to CRC diagnosis is associated with improved survival among CRC patients.

**Electronic supplementary material:**

The online version of this article (doi:10.1186/s12916-015-0332-5) contains supplementary material, which is available to authorized users.

## Background

The number of colorectal cancer (CRC) survivors is increasing thanks to early detection of tumors and advanced treatments [1]. Tumor characteristics at diagnosis are still the main determinants of survival, although there is a large variation in survival among patients with the same tumor stage and grade, and similar access to treatment. It has been hypothesized that lifestyle factors before and after diagnosis could influence this variability in survival [2].

There are only a small number of studies evaluating the potential role of lifestyle factors on CRC survival. Although results arising from these studies are not always consistent, most studies point towards a beneficial association of higher levels of physical activity [2-5], a healthy body weight [2,4,6,7], and consumption of dietary patterns low in red meat with CRC survival [7-10].

Given the lack of conclusive evidence on which lifestyle factors may determine survival, the World Cancer Research Fund/American Institute of Cancer Research (WCRF/AICR) advice for cancer survivors is to follow the same recommendations on diet, physical activity, and body fatness formulated for cancer prevention [11]. Within the context of the European Prospective Investigation into Cancer and Nutrition (EPIC) study, we have constructed an index score to reflect concordance with the WCRF/AICR recommendations for cancer prevention (the WCRF/AICR score); a higher WCRF/AICR score was associated with a lower incidence of overall and CRC cancer [12], as well as with a lower risk of overall and cancer mortality [13], among EPIC participants free of cancer at baseline.

The aim of the present study was to evaluate the association between concordance with the WCRF/AICR recommendations for cancer prevention on diet, physical activity, and body fatness before diagnosis and CRC-related and all-cause mortality among EPIC participants diagnosed with CRC during follow-up. We also evaluated the independent association of each component of the WCRF/AICR score with mortality among CRC cases.

## Methods

### Study population, CRC ascertainment, and sample selection

CRC cases in this study were identified among participants from the EPIC cohort, a large prospective study with over 520,000 participants enrolled in 23 centres in Denmark, France, Germany, Greece, Italy, the Netherlands, Norway, Spain, Sweden, and the United Kingdom between 1992 and 1999. Approval for this study was obtained from the ethical review boards of the International Agency for Research on Cancer and from all local institutions where subjects had been recruited for the EPIC study. Written informed consent was obtained from all participants before joining the EPIC study. The EPIC study methods have been described in detail elsewhere [14,15].

Cancer incidence during follow-up was determined through record linkage with regional cancer registries (Denmark, Italian centres except Naples, the Netherlands, Norway, Spain, Sweden, and the United Kingdom; complete up to December 2006) or via a combination of methods, including the use of health insurance records, contacts with cancer and pathology registries, and active follow-up through study subjects and their next-of-kin (France, Germany, the Italian center of Naples, and Greece; complete up to June 2010). CRC cases were selected among participants who developed colon (C18.0-C18.7, according to the Tenth revision of the International Classification of Diseases, Injuries and Causes of Death (ICD-10)), rectum (C19-C20), and overlapping/unspecified origin tumors (C18.8 and C18.9).

A total of 4,701 CRC cases were identified. Case exclusions included 426 cases diagnosed with CRC after vital status censoring date, 172 cases with *in situ* or non-primary tumors in the colon, 144 non-adenocarcinoma or tumor of unknown morphology, 21 due to missing date of death or diagnosis, 74 within the extreme ranking (top and bottom 1%) of the ratio energy intake/energy requirement, 37 with missing data on anthropometry, 56 with missing data on diet, 338 with missing data on physical activity (including all participants from Norway), and 141 women with missing data on breastfeeding (including all women from Bilthoven). The final sample included 3,292 CRC cases (1,497 men and 1,795 women; 2,071 colon cancer cases and 1,221 rectal cancer cases).

### Exposure assessment: data collection and dietary questionnaires

At recruitment (between 1992 and 1999), before cancer diagnosis, volunteers participating in EPIC filled out medical, dietary, and lifestyle questionnaires, including questions on alcohol use, smoking status, physical activity, education, reproductive history, breastfeeding, exogenous hormones use, and previous illnesses. Body weight and height were measured in all centres except Oxford (health conscious population) and France, where anthropometry was self-reported [16]. Usual food intakes were measured using country-specific validated dietary questionnaires [17] and individual nutrient intakes were derived from foods included in the dietary questionnaires through the standardized EPIC Nutrient Data Base [18]. To correct for any systematic under- or over-estimation of dietary intake between the study centres, a dietary calibration study was conducted. A random sample of 36,308 men and women (7.4% of the sample) completed a detailed computerized 24-h dietary recall, and nutrient intake was calculated using the standardized EPIC Nutrient Data Base [19]. Dietary exposures across centres were scaled using an additive calibration model [17]. Briefly, the difference between the sex- and center-specific mean of the values from the dietary questionnaire and the mean of the 24-h recall values was calculated and added to the questionnaire values. All dietary variables used in the present study were calibrated using additive calibration.

### WCRF/AICR score construction

A WCRF/AICR score, incorporating six of the WCRF/AICR recommendations for men (regarding body fatness, physical activity, food and drinks that promote weight gain, plant foods, animal foods, and alcoholic drinks) and seven for women (plus breastfeeding) was constructed. Detailed information on the operationalization of the score was previously published [12] and can be found in Additional file 1: Table S1. Briefly, we assigned, for each component, 1 point when the recommendation was met, 0.5 points when it was half met, and 0 points otherwise. When available, the quantitative criteria provided in the recommendations were used as cut-off points and intermediate cut-off points, defined by the authors, were used otherwise. For the recommendations, including several sub-recommendations (foods and drinks that promote weight gain or plant foods), the final score was the average of each sub-recommendation score (meaning that for these recommendations, plausible scores were 0, 0.25, 0.5, 0.75, and 1). Three recommendations were not implemented: i) the recommendation on preservation, processing, and preparation of foods because of insufficient data available, ii) the recommendation on dietary supplements which could not be operationalized in terms of cancer prevention without further assumptions about type or dose of supplementation, and iii) the special recommendation related to cancer survivors, who were advised to follow the same recommendations for cancer prevention. As the WCRF/AICR recommendations were not ranked according to priority, all major recommendations were summed to contribute equally to the total WCRF/AICR score. Therefore, the total WCRF/AICR score ranged from 0 to 6 for men and from 0 to 7 for women, with higher scores indicating greater concordance with the WCRF/AICR recommendations. The score was further categorized into four categories according to pre-defined cut-off points (0–2, 2.25–2.75, 3–3.75, and 4–6 points in men and 0–3, 3.25–3.75, 4–4.75, and 5–7 points in women).

### Outcome assessment: vital status ascertainment

Vital status follow-up was conducted by record linkage with regional and/or national mortality registries in all countries except France, Germany, Greece, and the Italian center of Naples, where data are collected through an active follow-up. Censoring dates for complete follow-up were between June 2005 and June 2009 in Denmark, the Netherlands, Spain, the United Kingdom, Sweden, and Italian centres except Naples. In Germany, Greece, France, and Naples, follow-up was based on a combination of methods, including health insurance records, cancer and pathology registries, and active follow-up through study subjects and their next-of-kin. In these centres, the end of follow-up was defined as the last known date of contact or the date of death, whichever came first. The last update of endpoint information occurred between December 2007 and December 2009.

Mortality data were coded according to the ICD-10. Up to six qualifiers of the cause of death were reviewed. The outcome of interest for the present study (death from CRC) was assigned based on the underlying cause of death [20].

### Statistical analyses

Cox proportional hazards regression was used to estimate the association between the WCRF/AICR score and death from CRC (primary endpoint) or death from any cause (secondary endpoint). Age was used as the primary time variable, with entry time defined as the subject’s age at CRC diagnosis and exit time as age censoring or death. All analyses were stratified by country to control for country-specific effects such as follow-up procedures and questionnaire design. The WCRF/AICR score was assessed as a continuous variable (1-point increment) and as a categorical variable (using the four pre-defined categories). The WCRF/AICR categorical variable was scored from 1 to 4, and trend tests were calculated on these scores. Multivariable models were adjusted for sex, year of CRC diagnosis, educational level (coded as no education, primary school, technical school, secondary school, university degree, and unknown/missing), smoking status (never, former, smoker, missing), tumor site (colon, rectum), tumor grade (well differentiated, moderately differentiated, poorly/undifferentiated, missing), and tumor stage (I, II, III, IV, missing); based on a previously described harmonization procedure among different EPIC centres [20]. Models were further adjusted for ‘lag time’ (years between recruitment/exposure assessment and CRC diagnosis), with no change in results; therefore, this variable was not included in the final multivariable models.

Sensitivity analyses were performed excluding participants who died within 6 months of CRC diagnosis and excluding participants with incomplete CRC stage data. Potential effect modifications by sex, mean age at diagnosis (<64.6 years vs. ≥64.6 years), year of diagnosis (1992–2001 vs. 2002–2008), median ‘lag time’ (<6.5 years vs. ≥6.5 years), tumor stage (I, II, III, IV), tumor site (colon vs. rectum), colon tumor sub-site (proximal vs. distal), and smoking status (former smokers, current smokers, and never smokers) were explored by modelling interaction terms (cross-products) between these variables (categorically) and the WCRF/AICR score (continuous), and conducting stratified analyses. Potential heterogeneity between countries in the association between the WCRF/AICR score and CRC-related mortality and overall mortality was assessed by calculating country-specific estimates and using random-effect meta-analyses (*I*^2^).

We estimated the independent association of each component of the WCRF/AICR score with CRC-related and overall mortality, after adjusting for all other components of the score. Finally, we evaluated the relative importance of each of the components of the WCRF/AICR score on CRC-related and overall mortality by subtracting alternately one component at a time from the original score, and including this component as a covariate in the model. To be able to compare the risk estimates to that of the total WCRF/AICR score, these alternative scores were assessed as continuous variables per 1 SD increments.

All statistical analyses were conducted using STATA 11 (StataCorp).

## Results

Of the 3,292 CRC cases from the EPIC cohort included in this study, 1,113 died after an average of 4.2 (SD, 3.3) years; 872 deaths were related to CRC. The average time between recruitment/exposure assessment and CRC diagnosis (‘lag time’) was 6.4 (3.3) years. The percentage of deaths was higher in individuals assigned to the lowest category of the WCRF/AICR score (those whose lifestyle was least concordant with the recommendations for cancer prevention) compared to those with higher WCRF/AICR scores. The percentage of CRC cases that were smokers at baseline and had a lower educational level was also higher in lower WCRF/AICR categories compared to the highest (Table 1).

**Table 1:** **Characteristics of the sample of CRC survivors by categories of WCRF/AICR score**

		**WCRF/AICR score categories**			
	**All participants**	**Category 1**	**Category 2**	**Category 3**	**Category 4**
Score range (men)/(women)	(0–6)/(0–7)	(0–2)/(0–3)	(2.25–2.75)/(3.25–3.75)	(3–3.75)/(4–4.75)	(4–6)/(5–7)
No. of participants	3,292	707	964	1,118	503
Age at recruitment, years [mean (SD)]	58.2 (7.6)	57.8 (7.2)	58.1 (7.6)	58.4 (7.6)	58.7 (8.7)
Age at CRC diagnosis, years [mean (SD)]	64.6 (8.0)	64.1 (7.6)	64.7 (7.8)	64.6 (7.8)	65.1 (9.0)
Follow-up time from recruitment to CRC diagnosis, years [mean (SD)]	6.4 (3.3)	6.3 (3.4)	6.6 (3.3)	6.2 (3.3)	6.3 (3.4)
Follow-up time from CRC diagnosis to end of follow-up, years [mean (SD)]	4.2 (3.3)	4.1 (3.3)	4.1 (3.2)	4.3 (3.4)	4.3 (3.3)
Deaths due to any cause [n (%)]	1,113 (33.8)	255 (36.1)	333 (34.2)	359 (32.1)	169 (33.6)
Deaths due to CRC [n (%)]	872 (26.5)	206 (29.1)	260 (27.0)	283 (25.3)	123 (24.5)
**Tumor stage [%]**					
I	18.3	19.7	18.4	18.3	16.3
II	18.5	19.2	17.8	18.3	19.5
III	26.2	24.9	28.5	25.8	24.6
IV	10.2	12.7	9.1	9.9	9.5
Unknown	26.7	23.5	26.1	27.7	30.0
**Tumor grade [%]**					
Well differentiated	7.3	8.8	7.9	6.2	6.8
Moderately differentiated	29.0	29.8	29.5	28.9	26.8
Poorly/Undifferentiated	7.6	6.7	8.0	8.6	5.8
Unknown	56.2	54.7	54.7	56.4	60.6
**Tumor site [%]**					
Colon – Proximal	28.9	28.0	28.1	28.2	33.0
Colon – Distal	28.7	30.4	28.8	28.9	25.5
Colon – Unspecified	5.4	4.8	5.7	5.6	5.0
Rectum	37.1	36. 8	37.3	37.3	36.6
**Sex [%]**					
Men	45.5	42.9	45.0	47.4	45.7
Women	54.5	57.1	55.0	52.6	54.3
**Educational level [%]**					
None	5.0	5.5	6.0	4.3	3.6
Primary school	32.8	33.8	34.1	34.8	24.7
Technical school	22.7	23.9	23.6	20.5	24.3
Secondary school	15.4	13.9	14.1	17.1	16.3
University degree	19.8	19.8	18.6	19.4	23.1
Not specified/Unknown	4.3	3.1	3.6	3.9	8.2
**Smoking status [%]**					
Never smokers	41.6	36.6	39.8	43.3	47.9
Former smokers	34.0	32.5	34.1	35.4	32.4
Current smokers	23.5	29.6	25.0	20.6	18.7
Unknown	1.0	1.3	1.0	0.7	1.0

The hazard ratios (HRs) (95% confidence intervals (CIs)) for CRC-related and overall mortality according to categories of the WCRF/AICR score are shown in Table 2. In multiple adjusted models, those within the second, third, and fourth categories of the WCRF/AICR score had a HR (95% CI) for CRC-related mortality of 0.87 (0.72–1.06), 0.74 (0.61–0.90), and 0.70 (0.56–0.89), respectively (*P* for trend <0.0001) compared to the first category (reference category); the corresponding HRs (95% CI) for overall mortality were 0.89 (0.75–1.06), 0.77 (0.66–0.92), and 0.79 (0.65–0.98) (*P* for trend 0.004). Similar HRs for CRC-related mortality were obtained in models run separately for men and women; however, the association between categories of the WCRF/AICR score and overall mortality was only significant in men (multivariable adjusted *P* for trend 0.004) but not in women (multivariable adjusted *P* for trend 0.242).

**Table 2:** **Hazard ratios (HR) and 95% confidence intervals (95% CIs) for CRC-related and overall mortality among CRC survivors according to categories of the WCRF/AICR score**

	**WCRF/AICR score categories**				
	**Category 1**	**Category 2**	**Category 3**	**Category 4**	***P*** **trend**
Score range (men)/(women)	(0–2)/(0–3)	(2.25–2.75)/ (3.25–3.75)	(3–3.75)/(4–4.75)	(4–6)/(5–7)	
**All**					
**CRC-related mortality**					
No. of deaths (%)	206 (29.1)	260 (27.0)	283 (25.3)	123 (24.5)	
Age- and country-adjusted^a^					
HR (95% CI)	1.00 (reference)	0.92 (0.76–1.11)	0.77 (0.64–0.93)	0.72 (0.57–0.91)	0.001
Multivariate adjusted^b^					
HR (95% CI)	1.00 (reference)	0.87 (0.72–1.06)	0.74 (0.61–0.90)	0.70 (0.56–0.89)	<0.0001
**Overall mortality**					
No. of deaths (%)	255 (36.1)	330 (34.2)	359 (32.1)	169 (33.6)	
Age- and country-adjusted^a^					
HR (95% CI)	1.00 (reference)	0.94 (0.79–1.10)	0.79 (0.67–0.93)	0.79 (0.65–0.97)	0.002
Multivariate adjusted^b^					
HR (95% CI)	1.00 (reference)	0.89 (0.75–1.06)	0.77 (0.66–0.92)	0.79 (0.65–0.98)	0.004
**Men**					
**CRC-related mortality**					
No. of deaths (%)	94 (31.0)	122 (28.1)	130 (24.5)	62 (27.0)	
Age- and country-adjusted^a^					
HR (95% CI)	1.00 (reference)	1.01 (0.77–1.34)	0.80 (0.60–1.05)	0.79 (0.56–1.12)	0.050
Multivariate adjusted^b^					
HR (95% CI)	1.00 (reference)	0.92 (0.69–1.22)	0.74 (0.54–0.96)	0.69 (0.48–0.99)	0.009
**Overall mortality**					
No. of deaths (%)	118 (38.9)	162 (37.3)	164 (30.9)	87 (37.8)	
Age- and country-adjusted^a^					
HR (95% CI)	1.00 (reference)	1.00 (0.79–1.28)	0.76 (0.59–0.97)	0.78 (0.58–1.06)	0.014
Multivariate adjusted^b^					
HR (95% CI)	1.00 (reference)	0.94 (0.73–1.21)	0.72 (0.56–0.93)	0.71 (0.52–0.97)	0.004
**Women**					
**CRC-related mortality**					
No. of deaths (%)	112 (27.7)	138 (26.0)	153 (26.0)	61 (22.3)	
Age- and country-adjusted^a^					
HR (95% CI)	1.00 (reference)	0.86 (0.66–1.11)	0.77 (0.60–0.99)	0.67 (0.48–0.92)	0.008
Multivariate adjusted^b^					
HR (95% CI)	1.00 (reference)	0.81 (0.62–1.06)	0.75 (0.57–0.97)	0.72 (0.51–1.00)	0.027
**Overall mortality**					
No. of deaths (%)	137 (33.9)	168 (31.7)	195 (33.2)	82 (30.0)	
Age- and country-adjusted^a^					
HR (95% CI)	1.00 (reference)	0.88 (0.70–1.11)	0.83 (0.67–1.05)	0.78 (0.59–1.03)	0.062
Multivariate adjusted^b^					
HR (95% CI)	1.00 (reference)	0.86 (0.68–1.09)	0.82 (0.65–1.04)	0.88 (0.66–1.18)	0.242

^a^Cox regression model, with age at CRC diagnosis as entry time and age at death or censoring as exit time, stratified by country; ^b^Multivariable adjusted models were further adjusted for year of CRC diagnosis, tumor stage, tumor grade, tumor site, sex, educational level, and smoking status.

A one point increment in the WCRF/AICR score was associated with a 10% (95% CI, 3–17%) and 7% (95% CI, 1–13%) risk reduction in CRC-related and overall mortality, respectively. Similar HRs for CRC-related and overall mortality associated with a 1-point increment in the score were obtained after performing sensitivity analyses. No evidence of effect modification by any of the analyzed variables (including sex) was detected (*P* for heterogeneity were all statistically not significant); however, the HRs for both CRC-related and overall mortality were lower among participants with less than 6.5 years of follow-up between recruitment and CRC diagnosis (compared with longer ‘lag time’), rectum cancer (compared to colon cancer), distal colon cancer (compared to proximal colon cancer), and never smokers (Table 3).

**Table 3:** **Hazard ratios (HR) and 95% confidence intervals (95% CIs) for CRC-related and overall mortality among CRC survivors associated with 1-point increment in the WCRF/AICR score; sensitivity and effect modification analyses**

	**CRC-related mortality**				**Overall mortality**			
	**No. of deaths (%)**	**HR** ^**b**^	**95% CI**	***P*** **for Heter.**	**No. of deaths (%)**	**HR** ^**b**^	**95% CI**	***P*** **for Heter.**
Age- and country-adjusted^a^	872 (26.5)	0.92	(0.86–0.98)		1,113 (33.8)	0.92	(0.87–0.97)	
Multivariate adjusted^b^	872 (26.5)	0.90	(0.83–0.97)		1,113 (33.8)	0.93	(0.87–0.99)	
**Sensitivity analyses** ^**c**^								
Excluding deaths within 6 mo of diagnosis	717 (24.4)	0.89	(0.82–0.97)		858 (29.2)	0.92	(0.85–0.99)	
Participants with complete CRC stage data	618 (25.6)	0.88	(0.80–0.96)		783 (32.4)	0.91	(0.84–0.98)	
**Effect modification analyses** ^**c**^								
**By sex**				0.983				0.597
Men	408 (27.3)	0.89	(0.79–1.00)		531 (35.5)	0.90	(0.81–0.99)	
Women	464 (25.8)	0.91	(0.82–1.01)		582 (32.4)	0.96	(0.88–1.06)	
**By mean age at diagnosis**								
<64.6 years	425 (26.4)	0.89	(0.80–0.99)	0.985	515 (32.0)	0.91	(0.82–0.99)	0.553
≥64.6 years	447 (26.5)	0.92	(0.83–1.03)		598 (35.5)	0.97	(0.89–1.07)	
**By year of diagnosis**								
1992–2001	548 (33.5)	0.88	(0.80–0.96)	0.889	691 (42.2)	0.90	(0.82–0.98)	0.415
2002–2008	324 (19.6)	0.93	(0.82–1.06)		422 (25.5)	0.96	(0.86–1.08)	
**By median ‘lag time’** ^**d**^								
<6.5 years	550 (33.3)	0.85	(0.77–0.94)	0.251	687 (41.6)	0.88	(0.81–0.96)	0.094
≥6.5 years	332 (20.2)	0.96	(0.84–1.08)		426 (26.0)	0.99	(0.88–1.10)	
**By tumor stage**								
I	51 (8.5)	0.86	(0.62–1.20)	0.279	76 (12.6)	0.80	(0.61–1.04)	0.067
II	78 (12.8)	0.87	(0.68–1.12)		108 (17.7)	0.85	(0.69–1.06)	
III	294 (34.1)	0.87	(0.75–1.00)		369 (42.8)	0.94	(0.83–1.07)	
IV	195 (57.9)	0.86	(0.72–1.04)		230 (68.2)	0.85	(0.72–1.01)	
**By tumor site**				0.263				0.137
Colon	537 (25.9)	0.95	(0.86–1.05)		693 (33.5)	0.99	(0.90–1.07)	
Rectum	355 (29.1)	0.85	(0.75–0.97)		420 (34.4)	0.86	(0.77–0.96)	
**By colon sub-site**				0.490				0.168
Proximal	288 (30.3)	1.07	(0.92–1.25)		310 (32.6)	1.09	(0.95–1.24)	
Distal	246 (26.1)	0.81	(0.70–0.94)		301 (31.9)	0.84	(0.73–0.96)	
**By smoking status**				0.892				0.773
Never smoker	365 (26.7)	0.86	(0.77–0.97)		448 (32.7)	0.94	(0.84–1.05)	
Former smoker	286 (25.6)	0.91	(0.79–1.05)		372 (33.3)	0.89	(0.79–1.01)	
Current smoker	214 (27.6)	1.00	(0.86–1.16)		282 (36.4)	0.96	(0.84–1.10)	

^a^Cox regression model, with age at CRC diagnosis as entry time and age at death or censoring as exit time, stratified by country; ^b^Multivariable adjusted models were further adjusted for year of CRC diagnosis, tumor stage, tumor grade, tumor site, sex, educational level, and smoking status; ^c^Sensitivity and stratified analyses were based on multivariable adjusted model; ^d^‘Lag-time’ means years between recruitment and CRC diagnosis.

Similar risk reductions in CRC-related mortality were observed when each component of the WCRF/AICR score was removed from the score; however, when the body fatness component and the breastfeeding component were excluded, the association between the score and CRC-mortality was no longer significant (Table 4).

**Table 4:** **Hazard ratios (HR) and 95% confidence intervals (95% CIs) for CRC-related and overall mortality among CRC survivors associated with one standard deviation (SD) increment in the WCRF/AICR score after removing each component of the score at a time**

	**CRC-related mortality**		**Overall mortality**	
	**HR** ^**a**^	**95% CI**	**HR** ^**a**^	**95% CI**
WCRF/AICR score	0.90	(0.84–0.97)	0.93	(0.87–0.99)
WCRF/AICR score – Body fatness	0.93	(0.87–1.00)	0.96	(0.90–1.02)
WCRF/AICR score – Physical activity	0.92	(0.86–0.99)	0.94	(0.89–1.01)
WCRF/AICR score – Food promote weight gain	0.89	(0.83–0.96)	0.92	(0.87–0.98)
WCRF/AICR score – Plant foods	0.92	(0.85–0.99)	0.94	(0.89–1.01)
WCRF/AICR score – Animal foods	0.88	(0.82–0.94)	0.91	(0.86–0.97)
WCRF/AICR score – Alcohol	0.90	(0.84–0.96)	0.93	(0.87–0.99)
WCRF/AICR score – Breastfeeding^b^	0.94	(0.85–1.04)	0.98	(0.88–1.08)

^a^Cox regression model, with age at CRC diagnosis as entry time and age at death or censoring as exit time, stratified by country and adjusted for year of CRC diagnosis, tumor stage, tumor grade, tumor site, sex, educational level, smoking status, plus the component that has been removed from the score. ^b^In women only.

When the independent association between the components of the score and CRC-related and overall mortality was examined, it was observed that meeting the recommendations for body fatness and plant food consumption significantly reduced mortality risk among CRC survivors in mutually adjusted models, compared to those who did not meet the recommendation (Table 5). We further evaluated the association of individual components of the WCRF/AICR score with mortality in colon and rectal cancer cases separately; in these analyses, meeting the body fatness recommendation was associated with improved survival in colon cancer cases whereas meeting the plant food recommendation reduced mortality risk in rectal cancer cases (data not shown).

**Table 5:** **Mutually adjusted hazard ratios (HR) and 95% confidence intervals (95% CIs) for CRC-related and overall mortality among CRC survivors, associated with the components of the WCRF/AICR score**

	**CRC-related mortality**			**Overall mortality**		
	**HR** ^**a**^	**95% CI**	***P*** **for trend** ^**b**^	**HR** ^**a**^	**95% CI**	***P*** **for trend** ^**b**^
**Body fatness**						
0	1.00	(ref)	0.010	1.00	(ref)	0.006
0.5	0.88	(0.73–1.06)		0.84	(0.71–0.99)	
1	0.78	(0.64–0.95)		0.78	(0.66–0.94)	
**Physical activity**						
0	1.00	(ref)	0.069	1.00	(ref)	0.162
0.5	0.86	(0.70–1.06)		0.84	(0.69–1.01)	
1	0.87	(0.74–1.02)		0.91	(0.79–1.05)	
**Foods that promote weight gain**						
0	1.00	(ref)	0.683	1.00	(ref)	0.544
0.25	0.87	(0.60–1.26)		0.80	(0.58–1.10)	
0.5	0.93	(0.64–1.33)		0.88	(0.64–1.21)	
0.75	0.87	(0.59–1.28)		0.84	(0.60–1.17)	
1	1.03	(0.64–1.69)		0.98	(0.64–1.51)	
**Plant foods**						
0	1.00	(ref)	0.069	1.00	(ref)	0.119
0.25	0.73	(0.56–0.97)		0.76	(0.60–0.97)	
0.5	0.66	(0.51–0.86)		0.72	(0.60–0.91)	
0.75	0.79	(0.60–1.02)		0.86	(0.68–1.09)	
1	0.65	(0.49–0.87)		0.68	(0.53–0.89)	
**Animal foods**						
0	1.00	(ref)	0.156	1.00	(ref)	0.123
0.5	1.14	(0.97–1.35)		1.09	(0.94–1.26)	
1	1.12	(0.83–1.51)		1.19	(0.92–1.54)	
**Alcohol intake**						
0	1.00	(ref)	0.658	1.00	(ref)	0.726
0.5	1.00	(0.78–1.28)		1.00	(0.81–1.25)	
1	0.95	(0.79–1.14)		0.96	(0.82–1.12)	
**Breastfeeding** ^c^						
0	1.00	(ref)	0.089	1.00	(ref)	0.407
0.5	0.68	(0.53–0.88)		0.76	(0.60–0.95)	
1	0.80	(0.63–1.02)		0.91	(0.73–1.13)	

^a^Cox regression model, with age at CRC diagnosis as entry time and age at death or censoring as exit time, stratified by country and adjusted for year of CRC diagnosis, tumor stage, tumor grade, tumor site, sex, educational level, and smoking status. All components were mutually adjusted for each other. ^b^ *P* for trend was calculated by modelling components of the WCRF/AICR score as continuous variables. ^c^In women only.

There was some evidence for heterogeneity between countries in the association between the WCRF/AICR score and CRC-related mortality (*I*^2^ = 31.9%; *P* for heterogeneity = 0.163) and overall mortality (*I*^2^ = 48.1%; *P* for heterogeneity = 0.052) (Additional files 2 and 3).

## Discussion

Higher pre-diagnostic concordance with the WCRF/AICR recommendations on diet, physical activity, and body fatness for cancer prevention was associated with a lower risk of CRC-related and overall death among participants diagnosed with CRC in the EPIC cohort. Despite not being able to demonstrate that meeting cancer prevention guidelines after cancer treatment would improve survival, what this and previous studies [13] suggest is that having a healthy lifestyle in line with these recommendations may improve survival among healthy participants, and this benefit also stands in those who develop CRC.

Concordance with the recommendations on body fatness and plant food consumption assessed before diagnosis was associated with reduced mortality risk among CRC patients. Body fatness and plant food consumption are well known risk factors for CRC [21-23] and have been also related to improved survival in CRC patients [2,24]. The breastfeeding component was also associated with longer survival in women diagnosed with CRC. Breastfeeding has been associated with lower risk of developing breast cancer, ovarian cancer, type 2 diabetes, and metabolic syndrome [25]. To our knowledge, no previous study has reported a positive association between breastfeeding and CRC risk [26-28]. In a previous study, meeting this special recommendation was associated with lower mortality due to cancer and circulatory diseases among healthy EPIC participants [13]. Further research is needed to confirm this potential beneficial effect of breastfeeding in the mother.

In two previous studies conducted within the EPIC cohort, we evaluated the association of the WCRF/AICR score with total and CRC incidence, as well as with overall and cancer-specific mortality, among cancer-free participants at baseline [12,13]. One-point increment in the WCRF/AICR score was associated with a 5% (95% CI, 3%–7%) reduction in the incidence of total cancer and with a 12% (95% CI, 9%–16%) reduction in the incidence of CRC [12]. Furthermore, 1-point increment in the WCRF/AICR score was associated with a 13% (95% CI, 12%–14%) lower risk of total mortality and with a 9% (95% CI, 7%–11%) lower risk of cancer-specific mortality [13]. These findings suggest that meeting the WCRF/AICR recommendations for cancer prevention reduces cancer incidence and mortality among EPIC participants free of cancer at baseline, as well as mortality in EPIC participants that developed CRC during follow-up.

A previous study based on a different cohort evaluated the association between concordance with the WCRF/AICR recommendations using a similar score and survival among female cancer survivors; contrary to the present study, the score was constructed based on exposure data collected on average 8.6 years after cancer diagnosis [29]. The score was associated with lower risk of all-cause mortality among all cancer patients, and specifically breast cancer survivors. The association between the score and mortality among other cancer type survivors – including CRC – was not statistically significant; however, the number of CRC patients (n = 380) and the number of deaths among these patients (n = 82) was small. In that study, both the physical activity and dietary recommendations predicted a lower mortality risk; meeting the body fatness recommendation (i.e., having a healthy weight) after diagnosis was indeed associated with a higher risk of death among cancer survivors. This finding is in opposition to what is observed in EPIC and other studies that considered pre-diagnostic body fatness: in the present study, meeting the body fatness recommendation (i.e., having a healthy weight) before diagnosis was significantly associated with reduced mortality among CRC cases. In concordance with our results, a previous study carried out in the same CRC cases identified in the EPIC cohort, found that having either general obesity or abdominal obesity before diagnosis was associated with a higher risk of death [24]. This obesity paradox – normal weight cancer patients at diagnosis have worse survival than overweight or obese cancer patients – has been previously reported and probably reflects unintentional weight loss due to underlying worse health status.

Another study by Pelser et al. [7], found that a composite lifestyle score, based on a healthy diet, physical activity, body mass index, alcohol consumption, and smoking, measured pre-diagnosis, was associated with improved survival after diagnosis with CRC. Specifically, obesity was related to an increased risk of all-cause mortality among colon cancer cases, whereas having a healthy diet (based on a diet quality score, the HEI-2005) improved survival in rectal cancer cases. This is in agreement with our findings. Similar to our results, the Pelser study found evidence of effect modification by ‘lag time’ [7]; our results suggested that the association of a healthy lifestyle with mortality among CRC survivors tended to fade among those with longer time periods between exposure assessment and cancer diagnosis. This could be due to changes in lifestyle between recruitment and CRC diagnosis or between cancer diagnosis and end of follow-up, which were not accounted for in the present study.

Advantages of the present study include its prospective design, high follow-up rate, and the large number of incident CRC cases included, identified within a large and heterogeneous European population, with a diversity of dietary patterns and other lifestyle habits. Detailed information on multiple exposures and confounders was available. In addition, lifestyle habits measured before cancer incidence may represent long-term exposure to these factors.

Limitations of our study include the lack of data on cancer treatment; although treatment may not differ substantially within European country, year of diagnosis, and tumor stage – factors that were adjusted for in our models – differences in the conduct of therapeutic interventions could be due to individual factors and health care. Indeed, heterogeneity of findings between countries could be partly explained by differences in therapy. Data on tumor stage and grade was limited; we performed sensitivity analyses excluding individuals with missing data on these variables with no major changes in results. Another limitation is the fact that lifestyle habits may have changed after cancer diagnosis and we did not have data on such changes over time. There is some evidence indicating that some cancer patients tend to adopt healthier lifestyle habits after treatment; however, other studies found no considerable change in behavior [30-34]. Therefore, we cannot assume that adherence to cancer prevention guidelines was similar after cancer diagnosis in the participants of the present study. The time interval between exposure assessment and cancer diagnosis differed among participants, leading to differences in ‘lag time’; however, we performed sensitivity analyses adjusting for differences in ‘lag time’ with no substantial change in results. Finally, as many statistical tests were performed, we cannot rule out that some of the statistically significant findings were due to chance.

## Conclusions

In conclusion, our findings suggest that, beyond smoking, a lifestyle in concordance with the WCRF/AICR recommendations on diet, physical activity, and body fatness, as reported by patients years before diagnosis, can improve survival in patients diagnosed with CRC. As expected, classical risk factors for CRC such as plant food consumption and body fatness were associated with CRC survival. Further research is needed to ascertain whether adhering to these recommendations after cancer diagnosis have a similar effect on survival.

## Additional files

Additional file 1: Table S1. World Cancer Research Fund/American Institute of Cancer Research (WCRF/AICR) recommendations for cancer prevention and operationalization of the WCRF/AICR score in the European Prospective Investigation into Cancer and Nutrition (EPIC) study. Additional file 2: Figure S1. Country-specific hazard ratios (HR) and 95% confidence intervals (95% CIs) for colorectal cancer (CRC)-related mortality among CRC survivors, associated with 1-point increment in the WCRF/AICR score by country. Cox regression model, with age at CRC diagnosis as entry time and age at death or censoring as exit time, and adjusted for year of CRC diagnosis, tumor stage, tumor grade, tumor site, sex, educational level, and smoking status. Additional file 3: Figure S2. Country-specific hazard ratios (HR) and 95% confidence intervals (95% CIs) for overall mortality among colorectal cancer (CRC) survivors, associated with 1-point increment in the WCRF/AICR score by country. Cox regression model, with age at CRC diagnosis as entry time and age at death or censoring as exit time, and adjusted for year of CRC diagnosis, tumor stage, tumor grade, tumor site, sex, educational level, and smoking status.

## Abbreviations

AICR: American Institute for Cancer Research
CI: Confidence interval
CRC: Colorectal cancer
EPIC: European Prospective Investigation into Cancer and Nutrition
HR: Hazard ratio
ICD-10: Tenth revision of the International Classification of Diseases, Injuries and Causes of Death
WCRF: World Cancer Research Fund
