# Supplementary material for: Pre-diagnostic concordance with the WCRF/AICR guidelines and survival in European colorectal cancer patients: a cohort study
Source: BMC Med. 2015 May 7;13:107. doi: 10.1186/s12916-015-0332-5 (PMC4423114; doi:10.1186/s12916-015-0332-5)
Supplement: Additional file 1: Table S1. — World Cancer Research Fund/American Institute of Cancer Research (WCRF/AICR) recommendations for cancer prevention and operationalization of the WCRF/AICR score in the European Prospective Investigation into Cancer and Nutrition (EPIC) study. [file 12916_2015_332_MOESM1_ESM.pdf]

Appendix Table 1: World Cancer Research Fund / American Institute of Cancer Research (WCRF/AICR) recommendations for cancer prevention and operationalization of the WCRF/AICR score in the European Prospective Investigation into Cancer and Nutrition (EPIC) study

WCRF/AICR Recommendation	Personal recommendations	Operationalisation	Scoring
**1 - BODY FATNESS**<br>Be as lean as possible without becoming underweight	1a - Ensure that body weight through childhood and adolescent growth projects towards the lower end of the normal BMI range at age 21	*Insufficient data available*	**n.a.**
	1b - Maintain body weight within the normal range from age 21	BMI 18.5 – 24.9 kg/m^2	**1**
		BMI 25 – 29.9 kg/m^2	**0.5**
		BMI<18.5 or BMI >30 kg/m^2	**0**
	1c - Avoid weight gain and increases in waist circumference throughout adulthood	*Insufficient data available*	**n.a.**
**2 - PHYSICAL ACTIVITY**[1]<br>Be physically active as part of your everyday life	2a - Be moderately physically active, equivalent to brisk walking, for at least 30 minutes every day	Manual/heavy manual job, or >2 h/w of vigorous PA, or >30 min/d of cycling/sports[2]	**1**
		15 – 30 min/d of cycling/sports	**0.5**
		<15 min/d of cycling/sports	**0**
	2b - As fitness improves, aim for 60 minutes or more of moderate or for 30 minutes or more of vigorous, physical activity every day	*Insufficient data available*	**n.a.**
	2c - Limit sedentary habits such as watching television	*Insufficient data available*	**n.a.**
**3 - FOODS AND DRINKS THAT PROMOTE WEIGHT GAIN**[2,3]<br>Limit consumption of energy-dense foods; avoid sugary drinks	3a - Consume energy-dense foods sparingly	**ED**[4] ≤125 kcal/100g/d	**1**
		**ED** >125 - <175kcal/100g/d	**0.5**
		**ED** >175 kcal/100g/d	**0**
	3b - Avoid sugary drinks	Sugary drinks intake[5] = 0 g/d	**1**

		Sugary drinks intake ≤250 g/d	**0.5**
		Sugary drinks intake>250 g/d	**0**
	3c - Consume fast foods sparingly, if at all	*Insufficient data available*	**n.a.**
**4 - PLANT FOODS**[2,3]<br>Eat mostly foods of plant origin	4a - Eat at least five portions / servings (at least 400 g) of a variety of non-starchy vegetables and of fruits every day	F&V intake ≥400 g/d	**1**
		F&V intake 200 - <400 g/d	**0.5**
		F&V intake <200 g/d	**0**
	4b - Eat relatively unprocessed cereals (grains) and/or pulses (legumes) with every meal	Dietary fibre intake ≥25 g/d	**1**
		Dietary fibre intake 12.5- <25 g/d	**0.5**
		Dietary fibre intake <12.5 g/d	**0**
	4c - Limit refined starchy foods	*Insufficient data available*	**n.a.**
	4d - People who consume starchy roots or tubers as staples should also ensure sufficient intake of non-starchy vegetables, fruits, and pulses (legumes)	*Not applicable to this population*	**n.a.**
**5 - ANIMAL FOODS**[3]<br>Limit intake of red meat and avoid processed meat	5a **-** People who eat red meat to consume less than 500 g a week, very little if any to be processed	Red and processed meat <500 g/w and processed meat intake <3 g/d	**1**
		Red and processed meat <500 g/w and processed meat intake 3-<50 g/d	**0.5**
		Red and processed meat ≥500 g/w or processed meat intake ≥50 g/d	**0**
**6 - ALCOHOLIC DRINKS**<br>Limit alcoholic drinks	6a - If alcoholic drinks are consumed, limit consumption to no more than two drinks a day for men and one drink a day for women	Ethanol intake ≤20 g/d (♂)<br>Ethanol intake ≤10 g/d (♀)	**1**
		Ethanol intake >20-30 g/d (♂)<br>Ethanol intake >10-20 g/d (♀)	**0.5**

		Ethanol intake >30 g/d (♂)<br>Ethanol intake >20 g/d (♀)	**0**
**7 - PRESERVATION, PROCESSING, PREPARATION**<br>Limit consumption of salt. Avoid mouldy cereals (grains) or pulses (legumes)	7a - Avoid salt-preserved, salted or salty foods; preserve foods without using salt	*Insufficient data available*	
	7b - Limit consumption of processed foods with added salt to ensure an intake of less than 6g (2.4g sodium)/day	*Insufficient data available*	
	7c - Do not eat mouldy cereals (grains) or pulses (legumes)	*Insufficient data available*	
**8 - DIETARY SUPPLEMENTS**<br>Aim to meet nutritional needs through diet alone	8a - Dietary supplements are not recommended for cancer prevention	*Not applicable to this population*	
**S1 - BREASTFEEDING**<br>Mothers to breastfeed; children to be breastfed	S1a - Aim to breastfeed infants exclusively up to six months and continue with complementary feeding thereafter	Cumulative breastfeeding ≥ 6 m	**1**
		Cumulative breastfeeding >0-<6 m	**0.5**
		No breastfeeding	**0**
**S2 - CANCER SURVIVORS**<br>Follow the recommendations for cancer prevention	S2a - All cancer survivors to receive nutritional care from an appropriately trained professional	*Not applicable to this population*	
	S2b - If able to do so, and unless otherwise advised, aim to follow the recommendations for diet, healthy weight, and physical activity	*Not applicable to this population*	

Abbreviations (in alphabetical order): BMI (Body Mass Index); d (day); ED (Energy Density); F&V (Fruits & Vegetables); g (grams); h (hours); m (months); min (minutes); n.a. (not available); PA (Physical Activity); w (week).

[1]Variables selected to operationalize the recommendation on physical activity are those included in the Cambridge physical activity index (except for vigorous physical activity) that has been validated for its use in epidemiological studies.

[2]The score for recommendations 3 and 4 was the result of averaging the scores of each sub-recommendation.

[3]All dietary variables used for the construction of the WCRF/AIRC score were calibrated using additive calibration (except for alcohol and energy density).

[4]Energy density (ED) was calculated as energy (kcal) from foods (solid foods and semi-solid or liquid foods such as soups) divided by the weights (g) of these foods. Drinks (including water, tea, coffee, juice, soft drinks, alcoholic drinks and milk) were not included in the calculation.

[5]Sugary drinks included both soft-drinks and fruit and vegetable juices.

Legend to Appendix Figure 1: Hazard ratios (HR) and 95% confidence intervals (95% CIs) for CRC-related mortality among CRC survivors, associated with 1-point increment in the WCRF/AICR score by country.

Cox regression model, with age at CRC diagnosis as entry time and age at death or censoring as exit time, stratified by country and adjusted for year of CRC diagnosis, tumour stage, tumour grade, tumour site, sex, level of school and smoking status.

Legend to Appendix Figure 2: Hazard ratios (HR) and 95% confidence intervals (95% CIs) for overall mortality among CRC survivors, associated with 1-point increment in the WCRF/AICR score by country.

Cox regression model, with age at CRC diagnosis as entry time and age at death or censoring as exit time, stratified by country and adjusted for year of CRC diagnosis, tumour stage, tumour grade, tumour site, sex, level of school and smoking status;
