# Supplementary figures and images for: Pre-diagnostic concordance with the WCRF/AICR guidelines and survival in European colorectal cancer patients: a cohort study
Source: BMC Med. 2015 May 7;13:107. doi: 10.1186/s12916-015-0332-5 (PMC4423114; doi:10.1186/s12916-015-0332-5)

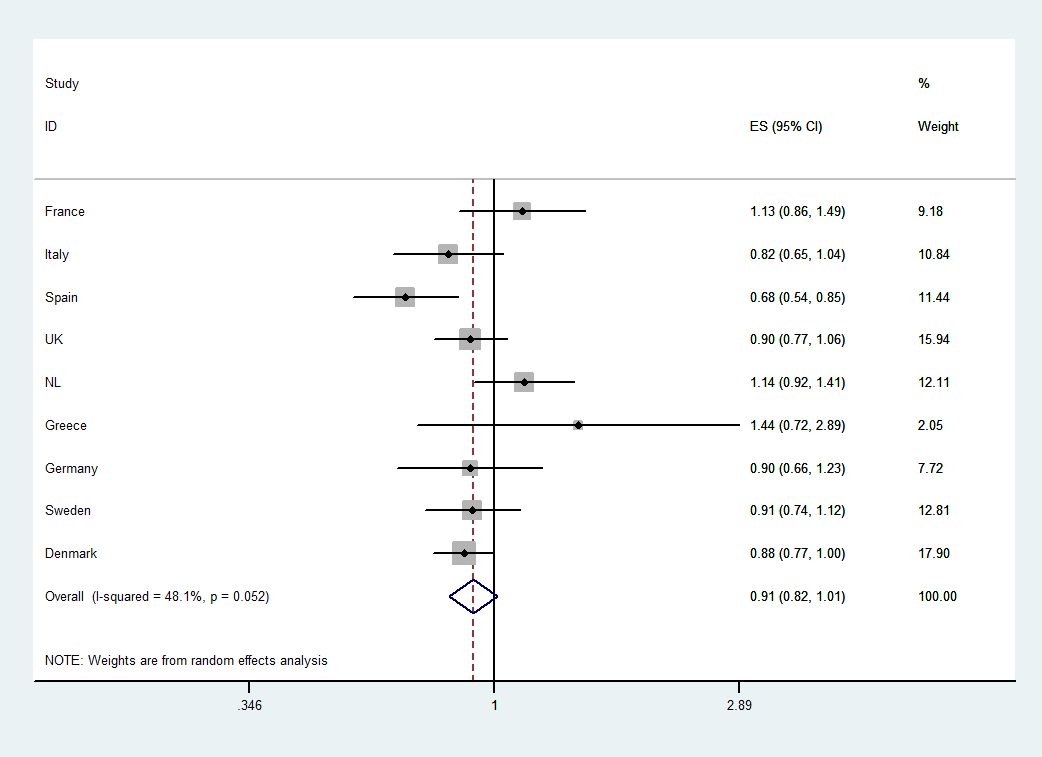

Supplement: Additional file 2: Figure S1. — Country-specific hazard ratios (HR) and 95% confidence intervals (95% CIs) for colorectal cancer (CRC)-related mortality among CRC survivors, associated with 1-point increment in the WCRF/AICR score by country. Cox regression model, with age at CRC diagnosis as entry time and age at death or censoring as exit time, and adjusted for year of CRC diagnosis, tumor stage, tumor grade, tumor site, sex, educational level, and smoking status. [file 12916_2015_332_MOESM2_ESM.tiff]

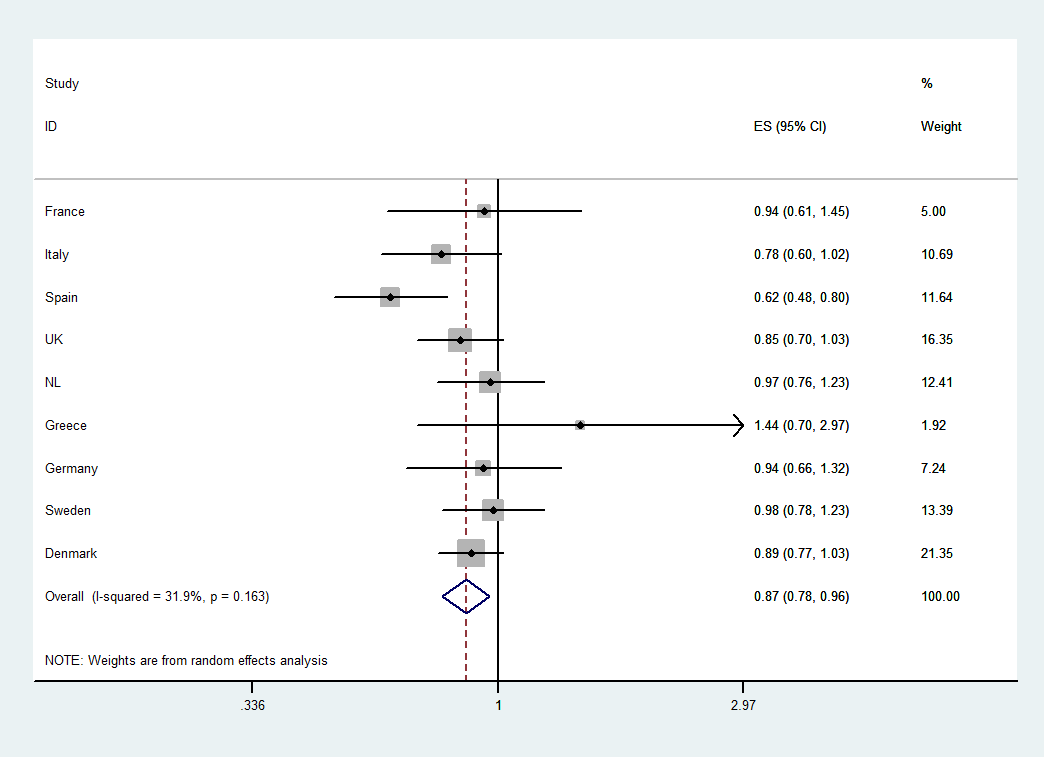

Supplement: Additional file 3: Figure S2. — Country-specific hazard ratios (HR) and 95% confidence intervals (95% CIs) for overall mortality among colorectal cancer (CRC) survivors, associated with 1-point increment in the WCRF/AICR score by country. Cox regression model, with age at CRC diagnosis as entry time and age at death or censoring as exit time, and adjusted for year of CRC diagnosis, tumor stage, tumor grade, tumor site, sex, educational level, and smoking status. [file 12916_2015_332_MOESM3_ESM.tiff]
